# Coupled analysis of arable land input intensity and output intensity based on sliding windows

**DOI:** 10.1016/j.mex.2024.102862

**Published:** 2024-07-23

**Authors:** Jiayi Jiang, Sijing Ye, Peichao Gao, Changqing Song

**Affiliations:** a Faculty of Geographical Science, Beijing Normal University, Beijing, 100875, China; b State Key Laboratory of Earth Surface Processes and Resource Ecology, Beijing Normal University, Beijing, 100875, China

**Keywords:** Sustainable intensification, Correlation analysis, Partial correlation analysis, Sliding window, Graphical user interface, Coupled analysis of arable land input intensity and output intensity based on sliding windows

## Abstract

Sustainable intensification (SI) of agriculture can produce more food to meet the demand of a growing population while considering ecosystem health. The current SI estimation framework ignores the complex coupling between input and output intensity of arable land. A method for coupled analysis of arable land input intensity and output intensity based on sliding windows is proposed. By calculating the correlation coefficient and partial correlation coefficient between input intensity and output intensity in different value ranges as the order parameter, the phase transition and the influence process of input intensity on output intensity can be explained. Meanwhile, a python-based framework is developed.

An application of the method was made to reveal the interaction process between annual provincial input intensity and output intensity in mainland China. Researchers in many fields may benefit from the method by obtaining a fast way to analysis the coupling relationship between driving and dependent variables in complex systems.

• New method for SI estimation is presented.
• The order parameter of the coupling relationship between input and output intensity is calculated based on sliding windows.
• Analysis of coupling relationships between driving and dependent variables in complex systems.

## Graphical abstract

**Figure:**
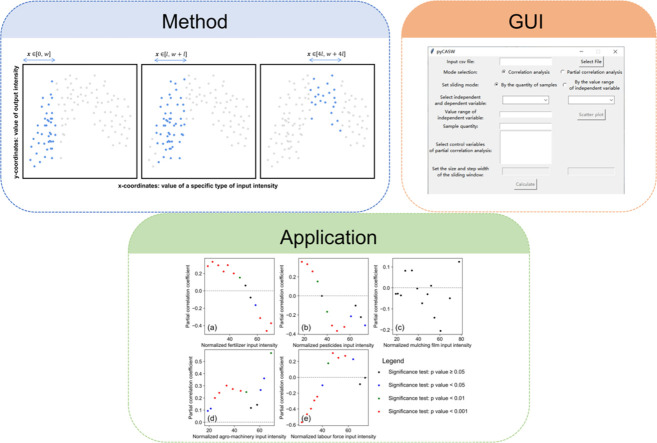

Specifications table

**Table:** 

Subject area:	Agricultural and Biological Sciences
More specific subject area:	Sustainable intensification
Name of your method:	Coupled analysis of arable land input intensity and output intensity based on sliding windows
Name and reference of original method:	Ye S, Wang J, Jiang J, Gao P, Song C. Coupling input and output intensity to explore the sustainable agriculture intensification path in mainland China. Journal of Cleaner Production. 2024;442:140,827. https://doi.org/10.1016/j.jclepro.2024.140827
Resource availability:	https://github.com/J-Jiang1124/pyCASW

## Background

The world is currently facing significant challenges in terms of food security, with nearly 700 million people still lacking access to basic food supplies [1]. To feed the continuously expanding population, there is a need for a substantial increase in food production [2]. However, agricultural development also needs to balance ecological health and stability [[3], [4], [5]]. Excessive expansion of farmland and the excessive use of fertilizers and pesticides have resulted in habitat destruction, loss of biodiversity, and climate change [[6], [7], [8], [9]]. Therefore, exploring a path towards sustainable agricultural development has become crucial for the future of all humankind. Sustainable intensification (SI) of agriculture is widely recognized as an important way to produce more food while considering ecosystem health, and it makes significant contributions to limiting the expansion of arable land, reducing pollution and conserving biodiversity [[10], [11], [12], [13], [14]].

The correlation between input intensity and output intensity is an essential aspect in numerous SI estimation frameworks [[15], [16], [17]]. In past study, the application of this correlation often involves efficiency metrics used to calculate ratio of output intensity to input intensity, such as: yield per unit input of energy, water and fertilizers; nitrogen use efficiency [18,19]. However, the effect of input intensity on output intensity may differ across various value ranges. This approach ignores the complex coupling relationship between input and output, which is frequently nonlinear, and cannot offer a quantitative explanation of how input intensity affects output intensity [15,20]. Besides, research in other fields provides references. The relationship between input and output needs to be evaluated in different dimensions [21,22].

In this study, a method for coupled analysis of input intensity and output intensity of arable land based on sliding windows is proposed. Based on Landau's theory of phase transition, the phase transition from gas to liquid comes along with the loss of symmetry, and the order parameter undergoes a sudden change, indicating that the system's state changes from disordered to ordered. The coupling relationship between input and output intensity in agricultural ecosystems can be seen as the extension and application of Landau's theory of phase transition. By calculating the correlation coefficient and partial correlation coefficient between input intensity and output intensity in different value ranges as the order parameter, the influence process of input intensity on output intensity can be explained. Meanwhile, a python-based framework for coupled analysis of input intensity and output intensity of arable land based on sliding windows (pyCASW) is developed. This method has been used to reveal the interaction process between annual provincial input intensity and output intensity in mainland China [23].

## Method details

**Fig. 1:**
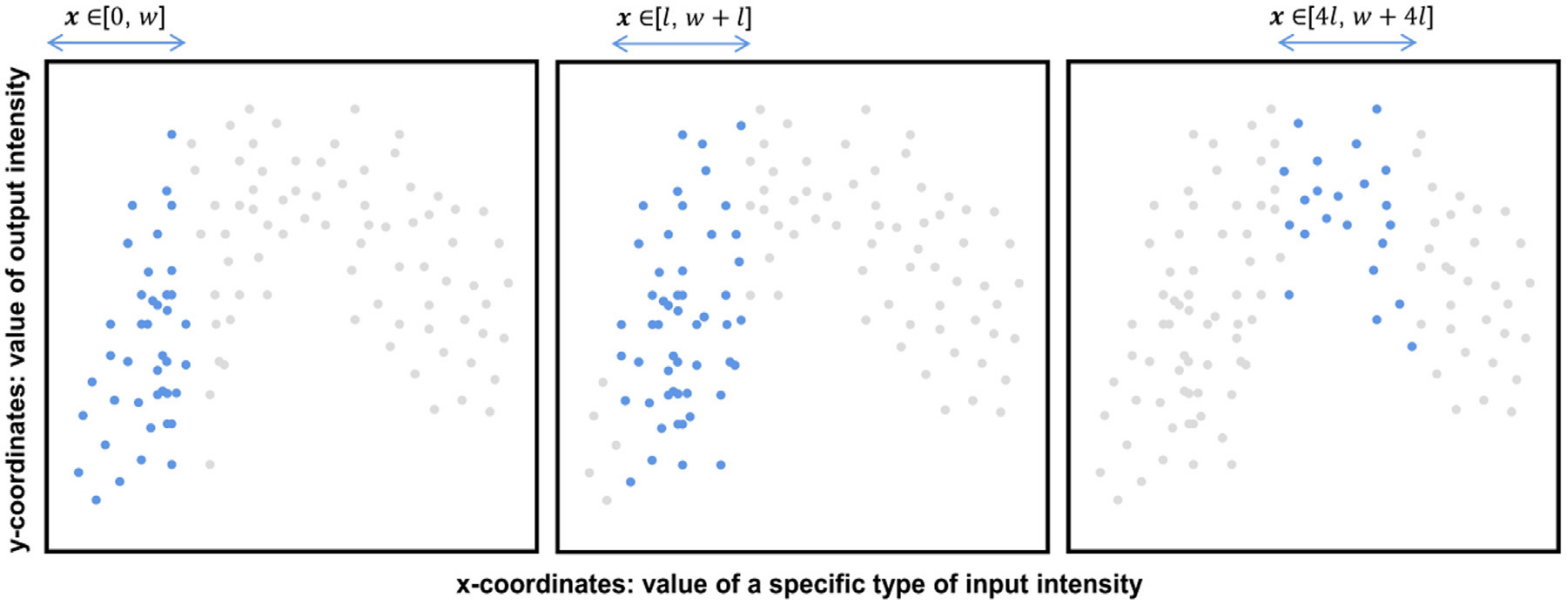
The procedure for calculating the correlation coefficient based on a sliding window by value range. The x-coordinate is the value of a specific type of input intensity (assuming the minimum value is 0). The y-coordinate is the value of the output intensity. (a)–(c) show the samples participating in the calculation within sliding windows of different value ranges.

To analyze the complex coupling relationship between input and output intensity of arable land, correlation coefficient is calculated in different value ranges by using a sliding window. The specific implementation process is shown in Fig. 1. The following are the steps (note: the input intensity and output intensity are suggested to be normalized to the range of 0–100 firstly).

Step 1, sort all sample values of a specific type of input intensity in ascending order.
Step 2, set the size and step width of the sliding window as w and l. The initial value range of the first sliding window is [0, w].
Step 3, extract samples of a specific type of input intensity whose values fall within the initial range (i.e., [0, w]). Calculate The correlation coefficient between the input intensity and output intensity. Output the result as the y-coordinate on a coordinate system, with the average value of the extracted samples of the specific type of input intensity set as the x-coordinate.
Step 4, shift the window by l. Repeat the calculation process in Step 3 with the value range of [l, w+l].
Step 5, continue to iterate through the calculation process outlined in Step 4 until the upper limit of the value range surpasses the maximum of the specific type of input intensity.

The pearson correlation coefficient and the partial correlation coefficient are used in the calculation process. The former can measure the overall correlation between two variables, while the latter can investigate the relationship between two variables while controlling for other variables.

**Fig. 2:**
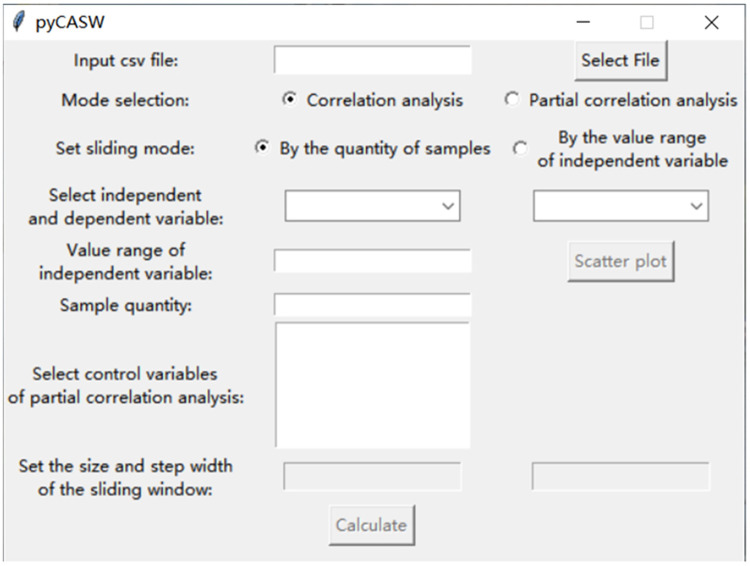
GUI was created in Tkinter to select the mode and setting the parameters easily.

A software application called pyCASW is designed for quick parameter setup and data analysis. A variety of Python libraries are used, including Numpy for mathematical calculations, Pandas for processing CSV data, Scipy and Pingouin for correlation analysis, Matplotlib for plots, and Tkinter for friendly user interface (see supplementary material for details). Both Windows and Mac OS support this software application. The graphical user interface (GUI) can be seen in Fig. 2.

**Fig. 3:**
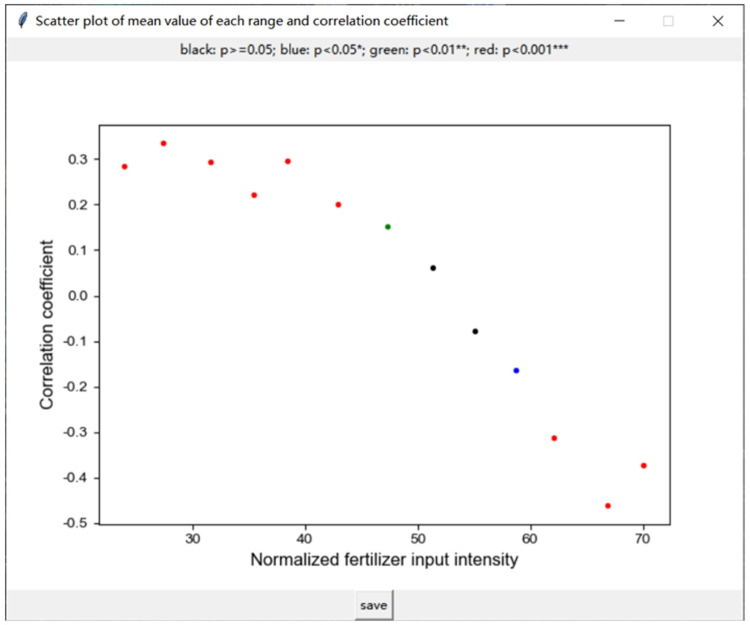
The scatter plot of the mean value of each range and correlation coefficient. The x-coordinate of the scatter plot is the mean value of each value range, and the y-coordinate is the correlation coefficient calculated in each value range between the value of a specific type of input intensity and output intensity. The points’ color of black, blue, green and red represent the four levels of p value, which are greater than or equal to 0.05, between 0.05 and 0.01, between 0.01 and 0.001, and less than 0.001. The x-coordinate is the value of a specific type of input intensity.

**Fig. 4:**
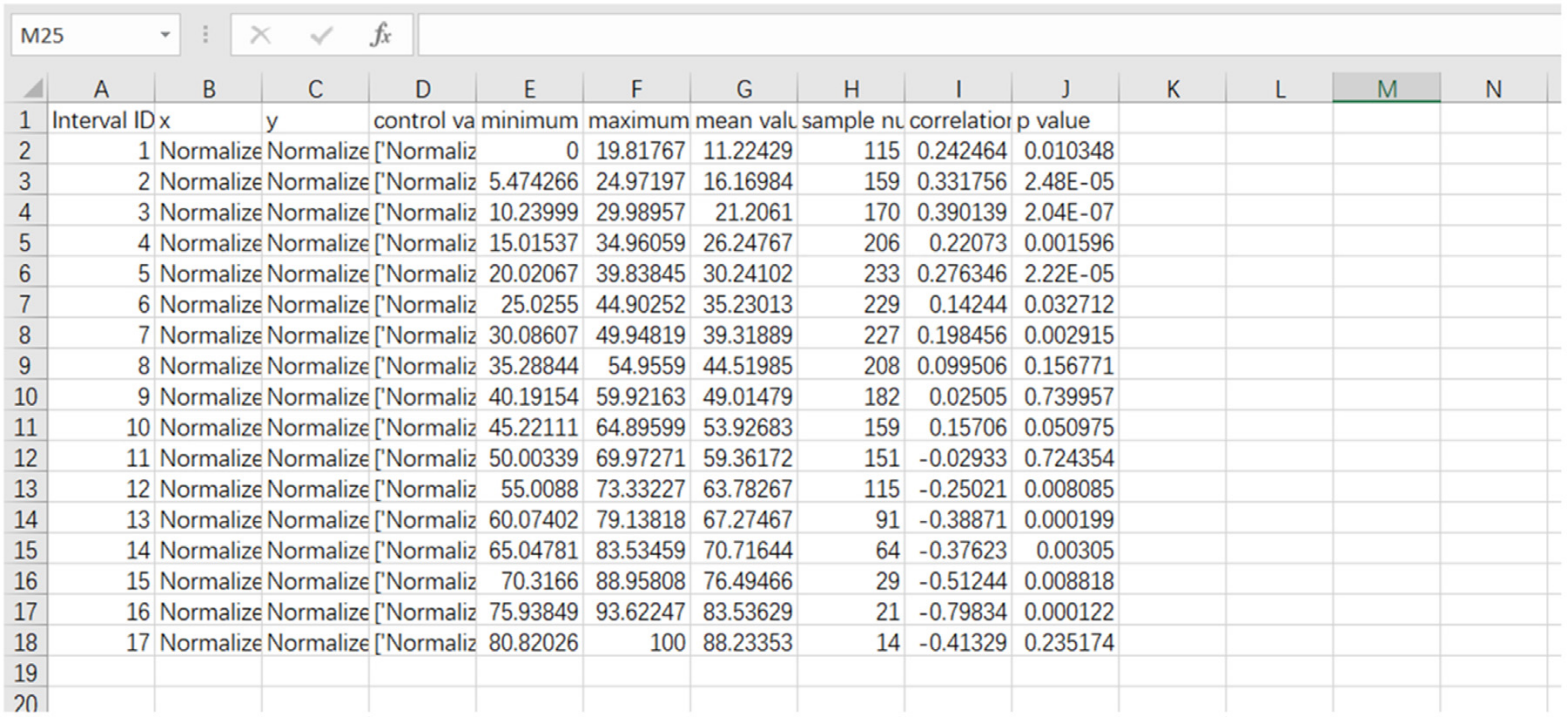
Example of exported CSV. Columns represent the ID of value range, x-coordinate, y-coordinate, control variables, minimum value of samples within the range, maximum value of samples within the range, mean value of samples within the range, samples number within the range, correlation coefficient and p value.

The users can set up the parameters in the GUI, including the independent variable, the dependent variable, control variables involved in the partial correlation analysis, the size and step width of the sliding window (see supplementary material for details). The scatter plot of the mean value of each value range and correlation coefficient can be drawn when the user clicks “Calculate”, which is similar to Fig. 3. The result of correlation and partial correlation analysis can be output as a csv file if the user clicks “save”, which is similar to Fig. 4. Scatter plots and csv files can be visualized to help explore the coupling relationship between input and output intensity and its phase transition.

## Method validation

The practicality and potential of the coupled analysis of arable land input intensity and output intensity based on sliding windows can be illustrated on a study of the coupling relationship between provincial output intensity of staple food grains and input intensity in China mainland during 1998–2019. The complete work has presented.

**Fig. 5:**
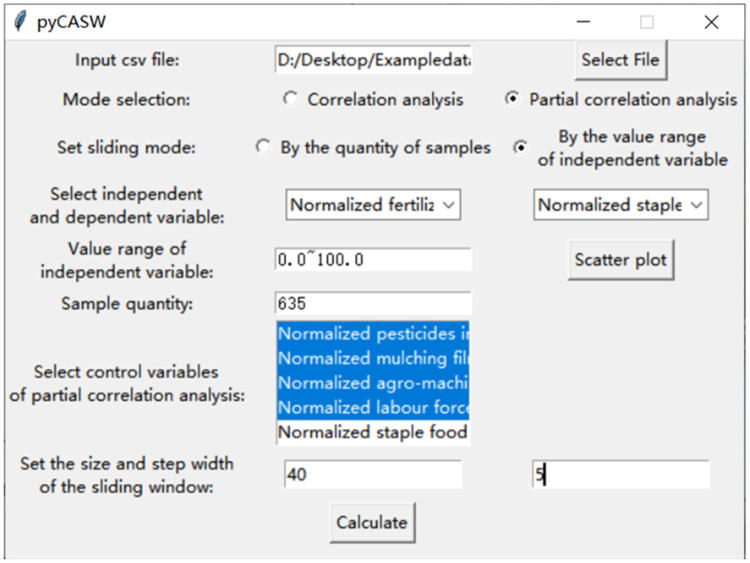
The parameter settings for conducting partial correlation analysis between normalized fertilizer input intensity and normalized staple food grains output intensity.

Fig. 5 shows the parameter settings for conducting partial correlation analysis between normalized input intensity and normalized staple food grains output intensity using pyCASW, taking the example of normalized fertilizer input intensity. Normalized pesticides input intensity, normalized mulching film input intensity, normalized agro-machinery input intensity and normalized labor force input intensity were chosen as control variables. After several attempts and trade-offs between the size of the sample size used for the partial correlation analysis and the precision of the analysis, the size of the sliding window was set to 40 and the step width was set to 5.

**Fig. 6:**
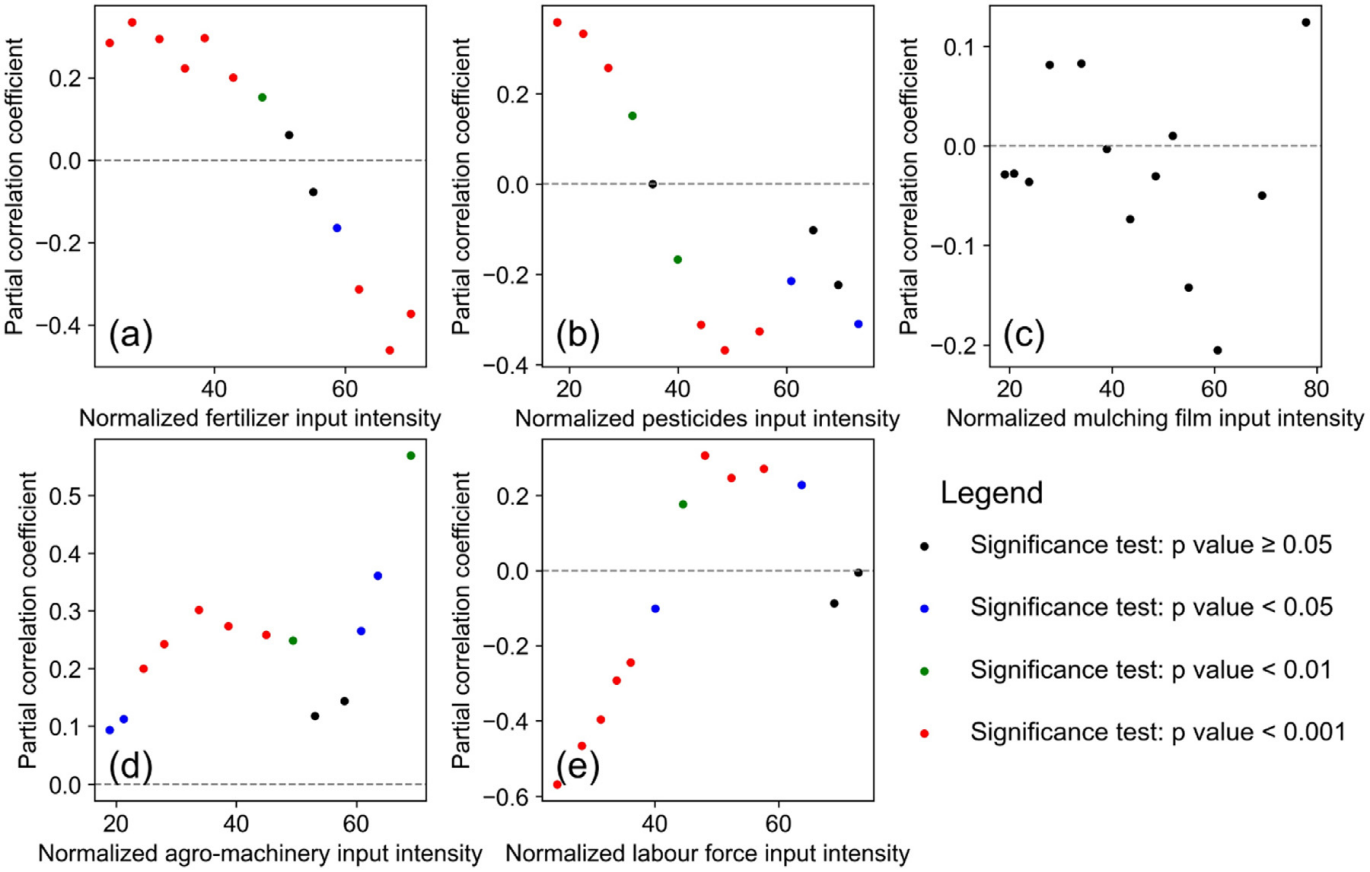
The result of partial correlation coefficients between normalized input intensity and normalized staple food grains output intensity based on sliding windows. (a-e) represent the influence process of normalized input intensity of fertilizer /pesticides/mulching film/agro-machinery/labor force respectively.

Fig. 6 shows the result of partial correlation analysis between normalized input intensity (fertilizer, pesticides, mulching film, agro-machinery, labor force) and normalized output intensity. The result uncovered how input intensity impacted output intensity. Taking normalized fertilizer input intensity as an example, normalized fertilizer input intensity at a low level showed a significant positive correlation with normalized staple food grains output intensity. With the increasing of normalized fertilizer input intensity, the significance level of this correlation became greater than 0.05. The transition first appeared in the sliding window of [35.288, 73.332]. As normalized fertilizer input intensity continued to increase, normalized fertilizer input intensity was significantly (*p* < 0.001) inversely correlated with normalized staple food grains output intensity. It demonstrated that excessive fertilizer inputs had hindered the staple food grains output in certain provinces. The proper fertilizer input intensity benefited for SI should not exceed the normalized threshold of 35.288, corresponding to 9.27 *E* + 14 sej/ha.

The coupled analysis of arable land input intensity and output intensity based on sliding windows offers a new method for analyzing the coupling relationship between input intensity and output intensity of arable land. The result can be used to estimate the suitability of input intensity and establish a threshold value for assessing the suitability of SI [23]. Moreover, the Python script called pyCASW can better serve research. The potential of this method is enormous, and researchers in many fields may benefit from it by obtaining a fast way to analysis the coupling relationship between driving and dependent variables in complex systems.

## Limitations

The method for coupling analysis of arable land input intensity and output intensity based on sliding windows used in this paper essentially analyzes the correlation coefficients and partial correlation coefficients within different value ranges. Therefore, this method suffers from the same limitations as correlation analysis. The details are as follows: (1) Effect of sample size: The size of the sample size affects the results of correlation analysis. Smaller sample size may lead to inaccurate results. The choice of the size of sliding windows needs to be carefully considered. (2) Effect of outliers: Outliers may have a significant effect on the correlation analysis. Outliers need to be dealt with before adopting this method. (3) Multicollinearity problem: If there is a high degree of correlation between the control variables, it may lead to inaccurate results of partial correlation analysis. The variables involved in the analysis need to be screened and processed

## Ethics statements

This work did not involve human subjects, animal experiments data, and data collected from social media platforms.

## CRediT authorship contribution statement

**Jiayi Jiang:** Methodology, Data curation, Visualization, Writing – original draft, Software. **Sijing Ye:** Conceptualization, Formal analysis, Methodology, Writing – original draft, Writing – review & editing. **Peichao Gao:** Methodology, Formal analysis, Project administration. **Changqing Song:** Conceptualization, Formal analysis, Supervision, Project administration, Funding acquisition.

## Declaration of competing interest

The authors declare that they have no known competing financial interests or personal relationships that could have appeared to influence the work reported in this paper.

## Data Availability

Data will be made available on request.

## Appendix Supplementary materials

Image, application 1

Image, application 2

Image, application 3

Image, application 4

Image, application 5
